# Translation and validation of the Hebrew version of the xerostomia inventory

**DOI:** 10.1007/s00784-026-06977-7

**Published:** 2026-06-19

**Authors:** Nitzan Aizenbud, Doron J. Aframian, Gabriel Nussbaum, Galit Almoznino

**Affiliations:** 1 In fulfillment of MSc. Thesis, Faculty of Dental Medicine, Hebrew University of Jerusalem, Jerusalem, Israel; 2 Faculty of Dental Medicine, Big Biomedical Data Research Laboratory, Dean’s Office, Hadassah Medical Center, Hebrew University of Jerusalem, Jerusalem, Israel; 3 Sjögren’s disease Center, Hadassah Medical Center, Jerusalem, Israel; 4 Department of Oral Medicine, Sedation & Maxillofacial Imaging, Hadassah Medical Center, Jerusalem, Israel; 5 The Institute of Biomedical and Oral Research, Faculty of Dental Medicine, The Hebrew University of Jerusalem, Jerusalem, Israel; 6 Big Biomedical Data Research Laboratory, Sjögren’s disease Center, Salivary Gland Impairment Laboratory and Clinic, Department of Oral Medicine, Sedation & Maxillofacial Imaging, Faculty of Dental Medicine, Hebrew University of Jerusalem Hadassah Medical Center, P.O. Box 12272, Jerusalén, 91120 Israel

**Keywords:** Salivary glands, Xerostomia, Dry mouth, Salivary gland dysfunction, Salivary flow

## Abstract

**Objectives:**

Xerostomia significantly affects oral health and quality of life, yet no validated Hebrew assessment exists. This study aimed to translate the Xerostomia Inventory (XI) into Hebrew (HXI) and evaluate its validity and reliability.

**Methods:**

The XI was translated into Hebrew using cross-cultural adaptation guidelines. The HXI was examined for internal consistency, using Cronbach’s alpha, as well as for construct validity (convergent and discriminant validity).

**Results:**

The HXI was completed by 102 xerostomia patients, 89.2% women, and the mean age was 63.5 ± 13.8 years with age range of 20–90 years. The mean HXI score was 39.9 ± 1.2, with score range 12–55. The HXI exhibited a high level of reliability with Cronbach’s α = 0.975. The convergent validity of the HXI, indicated by Spearman’s correlation between the HXI and the unstimulated salivary flow (USF) whole volume, demonstrated a strong, negative, and statistically significant correlation (*r* = -0.862). Strong negative correlations were also found between the total HXI score and other sialometry variables (ranging from *r*=-0.696 to *r*=-0.879). The absence of significant associations between the HXI scores and unrelated sociodemographic variables supports the discriminant validity of the HXI. Confirmatory factor analysis supported a robust unidimensional structure for the Hebrew 11-item HXI, with a single factor accounting for 80.3% of the total variance and all item loadings exceeding 0.82.

**Conclusions:**

The HXI is a valid and reliable instrument for assessing xerostomia in Hebrew-speaking populations, demonstrating psychometric robustness consistent with international versions.

**Clinical relevance:**

The HXI provides a valid and reliable tool for assessing patient-reported oral dryness, supporting diagnosis, treatment, and evaluation of interventions in Hebrew-speaking populations.

## Introduction

Saliva, often referred to as the “Aqua Vita” of the oral cavity, plays an essential role in maintaining oral health [1]. Xerostomia, the subjective sensation of dry mouth, is commonly used as a patient-reported outcome measure (PROM) of oral dryness [2]. Xerostomia may arise, albeit not exclusively, from diminished salivary secretion volume and/or alterations in saliva composition [3]. PROMs capture the perspectives of patients on their health status and the impact of disease and treatment on disability, lifestyle, and quality of life (QoL) [4]. PROMs are typically standardized, validated self-report questionnaires completed by patients or, when necessary, by proxies, providing clinicians with patient-centered evaluations of treatment/intervention effectiveness. Initially developed for research, PROMs are now widely used in clinical practice to assess provider performance and evaluate health policies and health promotion interventions [5]. These instruments are often administered at multiple time points, such as pre- and post-operatively, to document changes in overall health, QoL, and condition-specific outcomes [5]. One of the most widely used instruments for assessing oral dryness is the Xerostomia Inventory (XI) questionnaire. Developed in 1999 by Thomson et al., it provides a multi-item assessment of xerostomia-related symptoms [6]. The original English version demonstrated good internal consistency reliability, with a Cronbach’s alpha of 0.84 [6].

The aim of the current study was to translate the XI and determine the validity and reliability of the Hebrew XI (HXI). The XI was selected due to its high reliability and widespread clinical use for assessing subjective oral dryness [7, 8], and it has been validated in multiple languages, including Spanish, Greek, and Portuguese [9–11]. Validating the HXI would provide a standardized tool for clinical practice and for evaluation of public health improvement programs.

## Methods

### Study design and setting

This cross-sectional study analyzed medical records and response to the HXI of 102 patients suffering from xerostomia, who attended for treatment at the Salivary Glands Clinic, Department of Oral Medicine, Sedation and Maxillofacial Imaging, Faculty of Dental Medicine, The Hebrew University, Hadassah Medical Organization (HMO), Israel, between November 2021 and May 2022, and who met the inclusion and exclusion criteria.

### Ethical approval

This study was approved by the HMO Institutional Review Board (IRB) (No. 0655-21-HMO, date of approval 08/11/2021). The HMO IRB approved an informed consent, and all patients provided a signed informed consent with their signatures after receiving detailed explanations about the research.

### Study sample inclusion and exclusion criteria

#### Inclusion criteria

Native Hebrew speakers ≥ 18 years who attended the Salivary Glands Clinic, HMO with a primary complaint of xerostomia for more than three months.

#### Exclusion criteria

Those under 18 years, pregnant/breastfeeding women, patients with lack of judgment or with cognitive problem that cannot answer the questionnaire coherently, and patients that are not native Hebrew speakers.

### The XI translation process

The XI comprises 11 statements, and each one of them is scored from 1 to 5: (1) “Never”, (2) “Hardly ever”, (3) “Occasionally”, (4) “Frequently” and (5) “Always”. In this manner, the XI total score ranges from 11 (minimal XI score), to 55 (maximal XI score). The XI also has a “standard question” which gives another validity checking of the XI.

The translation and validation process included the following steps:

A. A “**forward/backward translation**” was conducted. For the “**forward**” translation, there were two bilingual translators with different backgrounds: the first translator was an oral medicine specialist (aware of the quantified concept and with clinical perspective), and the second translator was not aware of the quantified concept and without a clinical background. Both translators were native Hebrew speakers since the target language was Hebrew. They both made two independent “forward” translations of XI, from English to Hebrew.
B. A third, bilingual, independent translator compared and synthesized the two translations.
C. The united version was translated “**backward**” by two other independent, bilinguals, native English speakers, who have never been exposed to the original XI English version. One of them was a senior Bioscience researcher, and the second translator did not have any medical background. The two XI backward versions were performed from Hebrew back to English (the original XI language).
D. Subsequently, a discussion with a multi-disciplinary committee which included the translators, health professionals and oral medicine specialists was conducted to review and discuss the translation process and conclude on a pre-final Hebrew-version of XI.
E. The next step was the pilot study: the pre-final HXI was further tested on 10 xerostomia native Hebrew speaker patients, at the Salivary Glands Clinic.
F. Each of these patients had completed the HXI pilot and was then interviewed by the primary investigator about their impressions. If the HXI was not clear enough, the patient was asked to share thoughts and suggestions for corrections.

### Recruitment and assessment of patients

Patients who met the eligibility criteria for the study received an explanation of it and signed an informed consent form. The patients were recruited during the study period and all patients completed medical questioning, clinical examination, and sialometry. Collected data included age in years, sex (men/women), marital status, current work, collar, birth country, health-related habits, presence of any systemic morbidity, presence of salivary gland impairment related diseases, medications (including xerogenic) intake and liquids consumption. All co-morbidities, local conditions and medications intake were based on the medical information summary of referral letters from the general physician of the patient.

### Saliva collection

Sialometry included unstimulated salivary flow rate (USF) and stimulated salivary flow rate (SSF; 2% citric acid) measurements [12]. Sialometry was performed in standardized conditions as described previously [13]. Patients were instructed to avoid food, mouthwash, and tooth brushing for 2 h before the salivary flow measurement and to avoid salivary stimulants (sialagogues) on the day of the salivary flow measurement. USF and SSF were collected using the spitting method for 10 min: the patient sat comfortably on the dental unit chair, with eyes open, head tilted slightly forward and mouth pointing vertically downwards. It was explained to the patients that saliva is allowed to accumulate on the floor of the mouth when lips are closed, and the accumulated saliva should be split out into the test tube [13]. To collect the SSF a sterile applicator saturated with 2% citric acid was applied to the lateral margins of the tongue 5 times in 15 s interval and after 1 additional minute the SSF measurement was obtained using the spitting method for 10 min. We recorded:

1. The volume of USF whole salivary flow amount (USF whole amount), and its liquid fraction (USF liquid fraction), as well as foamy fraction (USF foamy fraction); and.
2. The volume of SSF whole salivary flow amount (SSF whole amount), and its liquid fraction (SSF liquid fraction), as well as the foamy fraction (SSF foamy fraction).

### Statistical analysis

IBM SPSS software No. 28.0 was used.

Descriptive statistics were presented by means and standard error (continuous variables), and absolute numbers and percentages (categorical variables). Explanatory statistics included reliability tests. The internal consistency was evaluated by calculation of Cronbach’s alpha. Cronbach’s alpha is a measure to evaluate reliability and is presented as a number between 0 and 1. A higher value of α indicates that the different items in the tested scale are measuring the same idea/concept. Cut-offs for α were as follows: α ≥ 0.9= “excellent”, 0.9 > α ≥ 0.8= “good”, 0.8 > α ≥ 0.7= “acceptable”, 0.7 > α ≥ 0.6= “questionable”, 0.6 > α ≥ 0.5= “poor”, 0.5 > α= “unacceptable” [14].

The construct validity of the HXI was evaluated by two sub-types of validity: the convergent validity and the discriminant validity. The convergent validity expresses the extent of two factors measuring the same construct [15]. In this study, it was expressed by the correlation between the HXI total score and USF, and also between the standard question and USF, that were both analyzed by Spearman’s correlation coefficient. The cut-offs of the Spearman’s correlation coefficient were as follows: −0.1 < *r* < 0.1: “none or very weak” correlation, −0.3 < *r*<−0.1 or 0.1 < *r* < 0.3: “weak” correlation, −0.5 < *r*<−0.3 or 0.3 < *r* < 0.5: “moderate” correlation, −1 < *r*<−0.5 or 0.5 < *r* < 1: “strong” correlation [16]. The Discriminant validity expresses low value of correlation between two measures of distinct contrast [17]. In this study, it was expressed by the correlation between the HXI total score and sociodemographic variables and was calculated by ANOVA (Analysis of variance) test.

The content validity represents the extent of relevance of items of an assessment tool in estimating a particular area. In this study it was evaluated by “content validity ratio” (CVR). CVR was measured by the experts rating each item based on its relevance: “Essential”, “Useful, but not essential” and “Not necessary”. The CVR was calculated using the formula: $\:CVR=\frac{\left({n}_{e}-\frac{N}{2}\right)}{\left(\frac{N}{2}\right)}$ (n_e_ is the number of experts indicating an item is “Essential” and N is the total number of panel experts). CVR values can range between 1 (perfect agreement) and − 1 (perfect disagreement). A value of 0 means that half of the experts in the panel agree that a certain item is essential.

The two-tailed statistical significance (α) level was defined as *p* < 0.05.

A Confirmatory Factor Analysis (CFA) was conducted to evaluate the underlying factor structure of the 11-item HXI. The analysis used Principal Axis Factoring (PAF) as the extraction method. Sampling adequacy was assessed using the Kaiser–Meyer–Olkin (KMO) measure, and Bartlett’s test of sphericity was used to evaluate the suitability of the correlation matrix. Factor extraction was determined by the Kaiser criterion (eigenvalues > 1) and visual inspection of the scree plot. Item retention was based on a factor loading cut-off of 0.40, following the recommendations of Hair et al. [18], to ensure significant contribution to the construct. While a Promax rotation was pre-specified, it is only applicable if multiple factors are identified.

## Results

### Results of the XI translation process

We performed the backward forward translation as depicted in steps A-C of the methods section. Subsequently, the multi-disciplinary committee (step D) reviewed and discussed all the translations versions: the original version, forward translation 1 version, forward translation 2 version, united version of forward translations 1 and 2, first and second backward translation, including semantic translation of the different rating options of XI and idiomatic phrases of the different 11-items and the standard question of XI. A consensus on pre-final Hebrew-version of XI was achieved regarding all questionnaire items and rating options by all committee members, in order to measure the different aspects of xerostomia.

In the next step the pilot study (step E) followed by an interview (step F) were both conducted. Only one patient of the pilot pre-test group suggested corrections for one component of the HXI. The final approved Hebrew version of the Xerostomia Inventory (HXI) is presented in the supplementary file.

One hundred and two xerostomic patients were recruited and assessed. Table 1 presents the socio-demographic, health-related habits, clinical and medical characteristics of the study sample. The study sample was predominantly female and middle-aged to old. The clinical profile was characterized by a high prevalence of systemic conditions, most notably Sjögren’s disease and rheumatoid arthritis, alongside common non-rheumatological comorbidities such as hypertension and hyperlipidemia. Consequently, the majority of the study sample were polymedicated, with a high frequency of xerogenic agent use.

**Table 1:** Sociodemographic, health-related habits, clinical and medical characteristics of the study sample (*N*=102)

Parameter	Variable			Number (%)
Sex	Women			91 (89.2)
	Men			11 (10.8)
Marital status	Married			69 (67.7)
	Divorced			13 (12.7)
	Widow			11 (10.8)
	Single			9 (8.8)
Current work	No			58 (56.8)
	Yes			44 (43.2)
Collar	White			22 (21.6)
	Blue			22 (21.6)
	Not working			58 (56.8)
Birth country	Israel			55 (53.9)
	Africa			15 (14.7)
	Asia			11 (10.8)
	Western Europe			7 (6.9)
	North America			7 (6.9)
	Eastern Europe			6 (5.9)
	South America			1 (1.0)
Health-related habits	Alcohol consumption			25 (24.5)
	Past smoking			21 (20.6)
	Current smoking			1 (1.0)
Rheumatological or autoimmune conditions	Sjögren’s disease			53 (52.0)
	Rheumatoid arthritis			23 (22.5)
	Fibromyalgia			18 (17.6)
	SLE			3 (2.9)
	Autoimmune hepatitis			2 (2.0)
	Osteoarthritis			1 (1.0)
	Other autoimmune disease			19 (18.6)
Other medical conditions	Hypertension			38 (37.3)
	Hyperlipidemia			35 (34.3)
	Gastroenterological disease			28 (27.5)
	Heart Disease			19 (18.6)
	I131 treatment			11 (10.8)
	Liver disease			9 (8.8)
	Diabetes			7 (6.9)
	General radiation			4 (3.9)
	Head and neck radiation			3 (2.9)
	Hepatitis C			2 (2.0)
	Other disease			58 (56.8)
Oncological conditions	Solid carcinoma			15 (14.7)
	Lymphatic cancer			3 (2.9)
	Hematological cancer			1 (1.0)
Salivary gland impairment related diseases	Drug induced xerostomia			53 (52.0)
	Chronic obstructive sialadenitis			19 (18.6)
	Sialadenosis			2 (2.0)
Medications	Systemic medications use			90 (88.2)
Parameter	Mean±SE		median	Minimum-maximum
Age (years)	63.51±1.36		65.50	20–90
Liquids consumption (ml)	1836.46±96.52		1500.00	100–6000
Number of medications	5.34±0.43	4.00		0–25
Number of xerogenic medications	0.7±0.1	1.0		0–5
USF (Whole volume, mL)	1.9±0.2	1.1		0–18.0
USF (Liquid fraction, mL)	1.3±0.2	0.9		0–12.0
USF (Foamy fraction, mL)	0.6±0.1	0.3		0–9.2
SSF (Whole volume, mL)	2.6±0.3	1.8		0–18.0
SSF (Liquid fraction, mL)	1.8±0.2	1.2		0–12.0
SSF (Foamy fraction, mL)	0.8±0.1	0.2		0–9.4

*SE* standard error of the mean

The difference between the USF and the SSF was statistically significant (Related-Samples Wilcoxon Signed-Rank Test: Z = 5.232, *p* value < 0.001, data are not shown in Table). The high range is attributed to a single woman participant diagnosed with chronic obstructive sialadenitis who exhibited an unusually high flow rate of 18 mL/10 min and was retained in the analysis to reflect the full clinical spectrum of the recruited patients. In the USF and in the SSF, most of the saliva was liquid.

The HXI was fully completed by all participants, with no exclusions required for missing data. The study sample exhibited a high symptomatic burden, as evidenced by a response distribution heavily weighted toward the higher end of the scale for both the total score and the global standard question (Table 2). While participants identified the physical sensation of oral dryness as the most severe symptom, the use of confectionery for relief was the least frequent behavior reported. Across the entire instrument, “Always” was the most prevalent selection while “Never” was the least utilized, reflecting the severe clinical nature of this specific sample. Specific mean values and response percentages for each item are detailed in Table 2.

**Table 2:** Descriptive statistics of the Hebrew Xerostomia Inventory (HXI)

Question	Options	Number (%)	Mean±SE
1. I sip liquids to aid in swallowing food	1-Never	9 (8.8)	3.6±0.1
	2-Hardly ever	17 (16.7)	
	3-Occasionally	15 (14.7)	
	4-Frequently	23 (22.5)	
	5-Always	38 (37.3)	
2. my mouth feels dry when eating a meal	1-Never	9 (8.8)	3.6±0.1
	2-Hardly ever	19 (18.6)	
	3-Occasionally	14 (13.7)	
	4-Frequently	23 (22.5)	
	5-Always	37 (36.3)	
3. I get up at night to drink	1-Never	7 (6.9)	3.6±0.1
	2-Hardly ever	15 (14.7)	
	3-Occasionally	24 (23.5)	
	4-Frequently	21 (20.6)	
	5-Always	35 (34.3)	
4. my mouth feels dry	1-Never	1 (1.0)	4.0±0.1
	2-Hardly ever	9 (8.8)	
	3-Occasionally	23 (22.5)	
	4-Frequently	24 (23.5)	
	5-Always	45 (44.1)	
5. I have difficulty in eating dry foods	1-Never	10 (9.8)	3.6±0.1
	2-Hardly ever	15 (14.7)	
	3-Occasionally	19 (18.6)	
	4-Frequently	21 (20.6)	
	5-Always	37 (36.3)	
6. I suck sweets or cough lollies to relieve dry mouth	1-Never	11 (10.8)	3.3±0.1
	2-Hardly ever	25 (24.5)	
	3-Occasionally	15 (14.7)	
	4-Frequently	22 (21.6)	
	5-Always	29 (28.4)	
7. I have difficulties swallowing certain foods	1-Never	10 (9.8)	3.5±0.1
	2-Hardly ever	13 (12.7)	
	3-Occasionally	24 (23.5)	
	4-Frequently	24 (23.5)	
	5-Always	31 (30.4)	
8. The skin of my face feels dry	1-Never	7 (6.9)	3.6±0.1
	2-Hardly ever	15 (14.7)	
	3-Occasionally	21 (20.6)	
	4-Frequently	22 (21.6)	
	5-Always	37 (36.3)	
9. My eyes feel dry	1-Never	6 (5.9)	3.7±0.1
	2-Hardly ever	12 (11.8)	
	3-Occasionally	24 (23.5)	
	4-Frequently	17 (16.7)	
	5-Always	43 (42.2)	
10. My lips feel dry	1-Never	5 (4.9)	3.6±0.1
	2-Hardly ever	15 (14.7)	
	3-Occasionally	22 (21.6)	
	4-Frequently	28 (27.5)	
	5-Always	32 (31.4)	
11. The inside of my nose feels dry	1-Never	9 (8.8)	3.5±0.1
	2-Hardly ever	20 (19.6)	
	3-Occasionally	12 (11.8)	
	4-Frequently	28 (27.5)	
	5-Always	33 (32.4)	
Standard question: How often does your mouth feel dry?	1-Never	0 (0.0)	3.9±0.1
	2-Hardly ever	12 (11.8)	
	3-Occasionally	22 (21.6)	
	4-Frequently	26 (25.5)	
	5-Always	42 (41.2)	
XI total score			39.9±1.2

The internal consistency presented by Cronbach’s alpha was 0.975 showing “excellent” reliability of the HXI.

Table 3 presents the inter-item correlation matrix in responses to the different questions of the HXI, including the standard question. There was a strong correlation observed among all the questions, with correlation coefficients ranging from 0.694 to 0.881.

**Table 3:** Inter-item correlation matrix between the different questions of the HXI, including the standard question

	1. I sip liquids to aid in swallowing food	2. My mouth feels dry when eating a meal	3. I get up at night to drink	4. My mouth feels dry	5. I have difficulty in eating dry foods	6. I suck sweets or cough lollies to relieve dry mouth	7. I have difficulties swallowing certain foods	8. The skin of my face feels dry	9. My eyes feel dry	10. My lips feel dry	11. The inside of my nose feels dry	Standard question: How often does your mouth feel dry?
1. I sip liquids to aid in swallowing food	1.000	0.874	0.804	0.751	0.857	0.694	0.824	0.791	0.795	0.740	0.868	0.769
2. my mouth feels dry when eating a meal	0.874	1.000	0.823	0.807	0.863	0.773	0.857	0.828	0.767	0.756	0.841	0.838
3. I get up at night to drink	0.804	0.823	1.000	0.710	0.810	0.769	0.823	0.765	0.775	0.744	0.849	0.788
4. my mouth feels dry	0.751	0.807	0.710	1.000	0.777	0.730	0.746	0.784	0.751	0.779	0.736	0.881
5. I have difficulty in eating dry foods	0.857	0.863	0.810	0.777	1.000	0.699	0.834	0.793	0.759	0.754	0.813	0.787
6. I suck sweets or cough lollies to relieve dry mouth	0.694	0.773	0.769	0.730	0.699	1.000	0.731	0.726	0.727	0.779	0.702	0.765
7. I have difficulties swallowing certain foods	0.824	0.857	0.823	0.746	0.834	0.731	1.000	0.772	0.765	0.765	0.808	0.812
8. The skin of my face feels dry	0.791	0.828	0.765	0.784	0.793	0.726	0.772	1.000	0.782	0.837	0.792	0.761
9. My eyes feel dry	0.795	0.767	0.775	0.751	0.759	0.727	0.765	0.782	1.000	0.800	0.819	0.813
10. My lips feel dry	0.740	0.756	0.744	0.779	0.754	0.779	0.765	0.837	0.800	1.000	0.735	0.800
11. The inside of my nose feels dry	0.868	0.841	0.849	0.736	0.813	0.702	0.808	0.792	0.819	0.735	1.000	0.773
Standard question: How often does your mouth feel dry?	0.769	0.838	0.788	0.881	0.787	0.765	0.812	0.761	0.813	0.800	0.773	1.000

We examined mean HXI scores across the four ordinal response categories of the standard dry-mouth question. The one-way ANOVA shown in Table 4 revealed a highly significant difference in HXI scores between these groups (F (3, 98) = 126.18, *p* < 0.001). Mean HXI scores were progressively higher in groups reporting greater frequencies of dryness, ranging from 19.6 for the “Hardly ever” group to 51.0 for the “Always” group (Table 4). Bonferroni-adjusted post-hoc tests demonstrated that all pairwise differences between categories were statistically significant (all *p* < 0.001).

**Table 4:** Mean HXI score by response to the standard question

Standard question: How often does your mouth feel dry?	N (%)	Mean HXI score (SD and 95% 95% Confidence Interval for Mean)				Post‑hoc Bonferroni
		Mean	Std. Deviation	95% Confidence Interval for Mean		
				Lower Bound	Upper Bound	
2-Hardly ever	12 (11.8)	19.6	5.6	16.0	23.1	Differs from 3, 4, 5 (*p*<0.001)
3-Occasionally	22 (21.6)	28.3	5.2	25.9	30.6	Differs from 2, 4, 5 (*p*<0.001)
4-Frequently	26 (25.5)	41.2	8.0	37.9	44.4	Differs from 2, 3, 5 (*p*<0.001)
5-Always	42 (41.2)	51.0	4.63	49.5	52.4	Differs from 2, 3, 4 (*p*<0.001)
Total	102 (100)	39.9	12.80	37.4	42.4	-

Oneway ANOVA Result: F (3, 98) = 126.18, *p* < 0.001; Effect size: η² = 0.794 (95% CI 0.716–0.835). Category 1 (“Never”) is excluded because no patients in this dataset selected that response

**Fig. 1:**
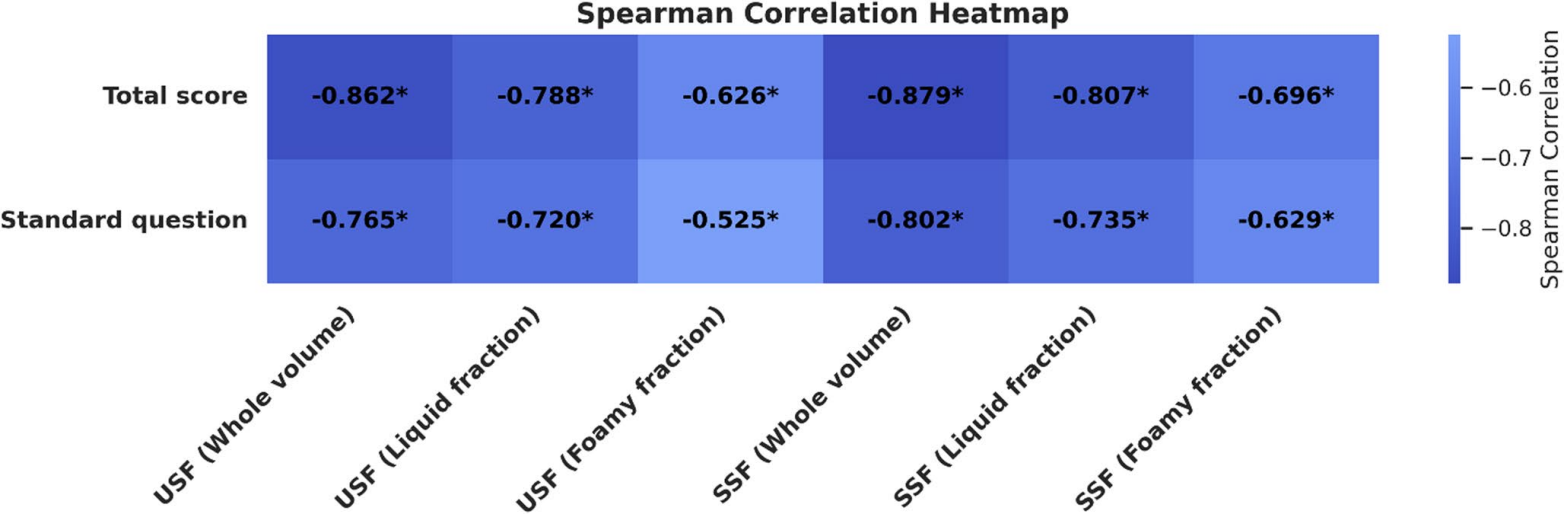
Convergent validity presented by correlation between the total HXI and the standard question scores and sialometry variables using Spearman’s correlation (*p<0.001)

The construct validity of the HXI was measured by convergent validity and discriminant validity. Figure 1 shows that the convergent validity of the HXI, indicated by Spearman’s correlation between the HXI and USF whole volume, demonstrated a strong and negative correlation. Strong correlations were also found between the total HXI score and other sialometry variables.

A strong, negative correlation was observed between the standard question and the USF. Additionally, strong, negative correlations were found between the standard question and all other sialometry variables.

As can be seen in Table 8 (Appendix), the discriminant validity confirmed there were no sociodemographic differences in the mean HXI scores, including all tested sociodemographic variables: sex, current work, collar, birth country, and parental birth countries.

Regarding the content validity, all the three experts in the panel, including the two oral medicine specialists and the senior bioscience researcher, agreed that all of the 11 items and the standard questions in the HXI were “essential”. According to the formula:

$CVR=\frac{\left(3-\frac{3}{2}\right)}{\left(\frac{3}{2}\right)}=1$, the HXI has perfect agreement (CVR=1).

**Fig. 2:**
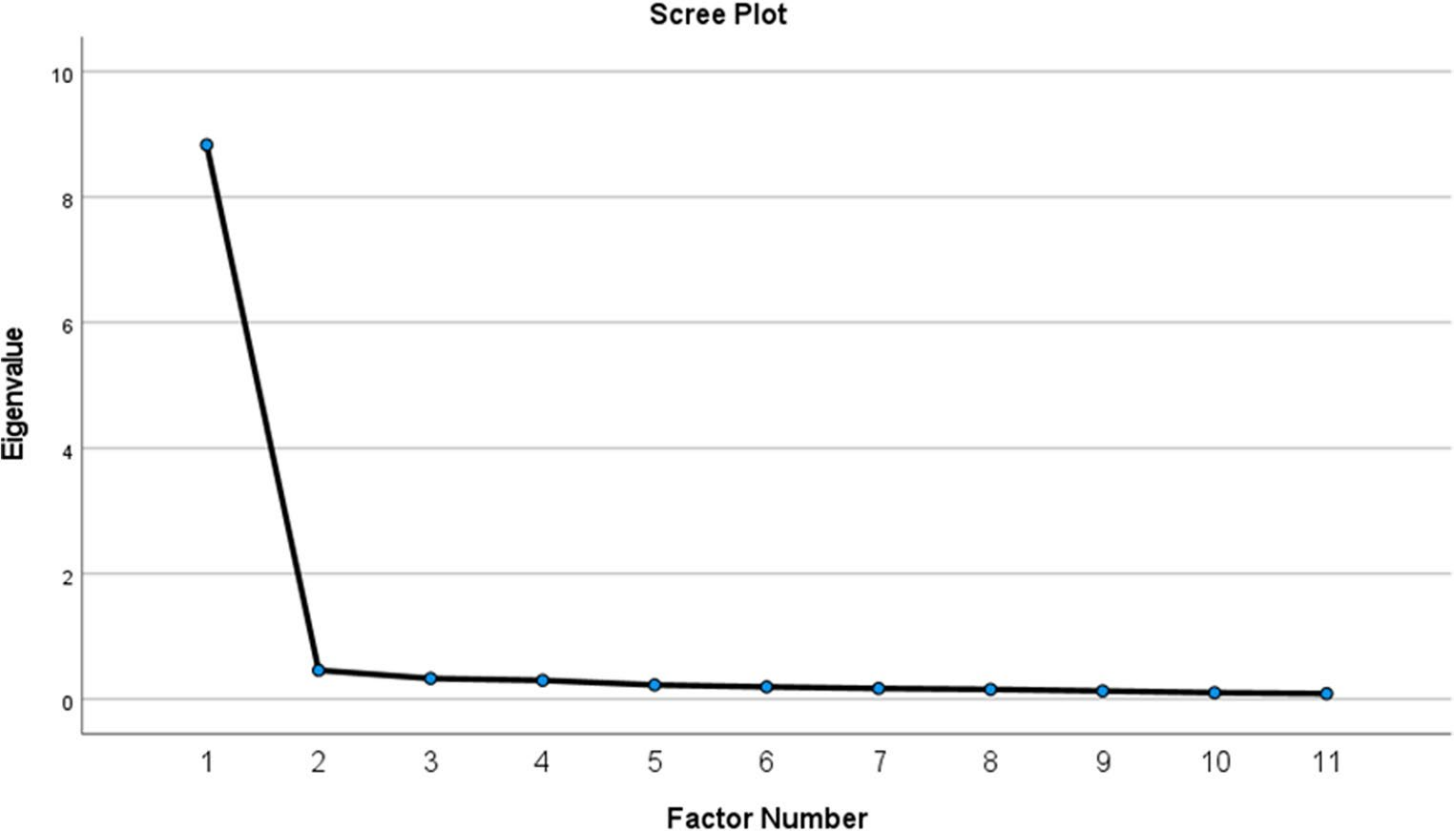
Scree plot of the Exploratory Factor Analysis for the HXI

The results of the factor analysis are shown in Table 5; Fig. 2. The sampling adequacy for the 11-item HXI was confirmed by a Kaiser–Meyer–Olkin (KMO) measure of 0.957, indicating an excellent distribution of values for factor analysis. Bartlett’s test of sphericity was highly significant (ꭓ^2^ (55) = 1385.419, *p* < 0.001), rejecting the null hypothesis that the variables were uncorrelated, and confirming the suitability of the data for factor extraction.

**Table 5:** Factor Loadings and Communalities for the HXI

Item number	hxi item	Factor loading	Communality (h2)
2	My mouth feels dry when eating a meal	0.930	0.866
1	I sip liquids to aid in swallowing food	0.907	0.823
11	The inside of my nose feels dry	0.902	0.814
5	I have difficulty in eating dry foods	0.902	0.814
7	I have difficulties swallowing certain foods	0.897	0.805
3	I get up at night to drink	0.890	0.793
8	The skin of my face feels dry	0.890	0.792
9	My eyes feel dry	0.873	0.763
10	My lips feel dry	0.866	0.750
4	My mouth feels dry	0.852	0.726
6	I suck sweets or lollies to relieve dry mouth	0.822	0.676

Confirmatory Factor Analysis (CFA) via Principal Axis Factoring (PAF) identified a clear unidimensional structure. Based on the Kaiser criterion, only one factor yielded an eigenvalue greater than unity (initial eigenvalue = 8.834), accounting for 80.3% of the total variance. As can be seen in Fig. 2, the Scree Plot exhibited a definitive “elbow” after the first component, with subsequent components contributing negligibly to the variance (all eigenvalues < 0.47). Detailed factor loadings and communalities are presented in Table 6. All 11 items displayed robust factor loadings, ranging from 0.822 (Item 6) to 0.930 (Item 2), well exceeding the conservative threshold of 0.40. Communalities (h^2^) ranged from 0.676 to 0.866, suggesting that the single-factor model explains a high proportion of the variance for each individual item and confirming the structural integrity of the Hebrew translation.

**Table 6:** Validity and reliability tests of translated XI to various languages

Language	Number of patients	Type of patients	XI score (Mean ±standard deviation)	Cronbach's α	ICC	Construct validity	Factor analysis	Other
Hebrew- current version	102	Xerostomia patients	Mean ±SE: 39.9±1.2	0.975	0.78	· Spearman: r= −0.862, *P* value <0.001. · Correlation between the standard question and USF: r=−0.765, *P* value <0.001.	1-Factor (80.4% variance explained)	"Cronbach's Alpha if Item Deleted" showed very high values (0.971–0.974), which were also similar to the original α, meaning that they were all required for the "excellent" reliability of the HXI.
German [22]	39	Radiation-induced xerostomia	44.2 ± 5.0	0.92	0.85	-	Confirmatory factor analysis showed good to moderate values	-
Spanish [10]	41	Xerostomia patients	40.8±10.0	0.89	0.59–0.91	Pearson: r= −0.32, *P* value= 0.050	Not provided	-
Portuguese [9]	30	Primary Sjögren's disease	42.2±8.9	0.90	0.79–0.94	Pearson: r= −0.781, P value= 0.010 for resting saliva flow and r=−0.757, P value= 0.010 for stimulated saliva flow	Not provided	-
Korean [25]	194	Primary Sjögren's disease	median 37; IQR 30–44	0.868	0.48–0.82	· Spearman: r= −0.447, *P* value<0.001 for resting salivary flow and r=−0.515, P value<0.001 for stimulated salivary flow. · Correlation between the standard question with USF: r= 0.772, *P* value<0.001	Not provided	-
Turkish [26]	69	Primary Sjögren's disease	36.41 ± 7.67	0.869	0.993	Spearman: r= −0.611, *P* value<0.001 for resting salivary flow and r= −0.674, *P* value<0.001 for stimulated salivary flow.	Not provided	"Cronbach's Alpha if Item Deleted" showed similar values to the Cronbach's α value, for all 11 XI questions, meaning no question can be deleted.
Greek [11]	100	Older greeks	Not provided	0.78	Not provided	Significant correlations with self-perceived dryness	7-item scale identified	4 items were deleted
French [23]	65	Older adults	20.0±7.4	0.79	0.83	No relation with salivary flow	1-Factor (32.7% variance explained)	-
Persian [27]	100		-	0.84	0.95	-	-	-
Thai [24]	200	Thai adults (50+)	Median 21.5, IQR 16–27	0.875	Not provided	Moderate to strong correlation with comparator instruments	1-Factor	

## Discussion

The findings of this study confirm that the Hebrew version of the XI is a robust and reliable instrument for assessing patient-reported oral dryness. The high internal consistency as indicated by Cronbach’s alpha, coupled with successful construct and discriminant validity, align with existing international versions. Furthermore, factor analysis supports a unidimensional structure, with a high explained variance of 80.3%, reinforcing the structural integrity of the HXI for use in Hebrew-speaking populations.

The findings from the pilot pre-test, in which only one patient suggested a correction, represent a feedback rate of less than 20%. This ensures that the HXI remains within the clarification guidelines established in the methodology [8].

Xerostomia has a significant impact on oral health, caries development, and QoL related to comfort, social interaction, swallowing and speech. However, objective measurement of salivary flow rate do not always correlate with the subjective xerostomia symptoms [19]. PROMs are utilized to evaluate health status of patients from their own perspective at a specific moment [4]. Therefore, they provide clinicians with an insight on the subjective general health and QoL perception of the patients, thus supporting a patient-centered approach to care [20].

Despite xerostomia being a reasonably prevalent condition [2, 21], no validated assessment exists in the Hebrew language. Linguistic validation of the Hebrew version of the XI would align with established international standards for the cross-cultural adaptation of PROMs. By documenting this validation in the international literature—joining existing adaptations such as the German [10, 22], Portuguese [9], Greek [11], French [23] and Thai [24] versions—we ensure the accessibility of the XI instrument to the global scientific and clinical communities. Such dissemination is fundamental for several reasons. First, it facilitates multicenter international trials by providing a standardized, validated instrument for diverse geographic samples. Second, it promotes methodological consistency, enabling researchers to benchmark data against global standards.

Ultimately, the publication of this Hebrew version contributes to the broader psychometric evidence base, confirming robustness of the instrument across varied linguistic and cultural contexts.

Table 6 provides a comparative analysis of the validity and reliability metrics obtained in this study for the HXI, juxtaposed with those from similar assessments conducted across various language translations, including German [22], Spanish [10], Portuguese [9], Korean [25], Turkish [26], Greek [11], French [23], Persian [27], and Thai [24]. To the best of our knowledge, this represents a comprehensive comparison of full-length (11 or 12-item) versions available in the English-language literature. The data on Table 6 represent specific study samples and are intended to show instrument consistency rather than representative population prevalence.

This analysis explicitly distinguishes between the full-length XI used in this study and the 5-item Summated version (SXI-5) derived from the original instrument [28]. The SXI-5 has been also validated in various languages such as Chinese [29], Indonesian [30], Arabic [31], Hindi [32], Dutch [28], German (validated alongside XI) [22] and Thai [24] (validated alongside the XI). While SXI instrument offers acceptable reliability and is useful for screening or large-scale studies, they differ in item composition, scoring, and dimensional structure compared with the full XI, and therefore does not capture the full range of xerostomia symptoms assessed by the original instrument. For this reason, our study focused exclusively on the full XI, including all 11 original items and the standard xerostomia question, to ensure methodological consistency and allow direct comparison with previous full-length validations.

The mean HXI score on the HXI was 39.9 ± 1.2, which is comparable to the scores obtained with the Portuguese (42.2 ± 8.9), Germen (44.2 ± 5.0), Spanish (40.8 ± 10.0), Korean (median 37; IQR 30–44), and Turkish (36.4 ± 7.7) versions. These versions also included patients with autoimmune conditions such as Sjögren’s disease. Scores obtained from the French (20.0 ± 7.4) and the Thai versions (median 21.5) that included clinical convenience samples of older adults but without autoimmune conditions were lower.

The robust inverse correlation between subjective data and sialometry (*r* = −0.862) establishes superior convergent validity, as it confirms the capacity of the instrument to yield scores that align significantly with an independent, objective criterion—sialometry—measuring the identical physiological construct. The high correlation between subjective data and sialometry is a direct result of the clinical profile and was also found in the Portuguese (*r*= −0.781), Korean (*r* = 0.772 with the standard question), and Turkish (*r*= −0.611) versions.

In accordance with the validation framework of the original 1999 study by Thomson et al.[6], the Hebrew HXI demonstrated excellent convergent validity (specifically concurrent validity) when tested against a single-item global standard. Our analysis revealed a progressive gradient in mean HXI scores across the four ordinal response categories of the standard dry‑mouth question (see Table 3B). In our data, all pairwise contrasts were statistically significant, and the effect size was large (η² = 0.794), indicating that the HXI discriminates well between individuals reporting different levels of xerostomia. This reinforces the utility of the HXI as a continuous, sensitive measure of xerostomia severity.

The HXI demonstrated exceptional internal consistency with a Cronbach’s alpha of 0.975. This value is the highest among all compared international versions, exceeding the original Thomson et al. (1999) validation (0.84) [6] as well as the German (0.92) [22], Portuguese (0.90) [9], and Spanish (0.89) [10] adaptations. The “Cronbach’s Alpha if Item Deleted” of the HXI remained stable between 0.971 and 0.974, proving that every item in the 12-item inventory is essential for maintaining its high reliability. Such high Cronbach’s alpha values imply that the items on the XI are highly interrelated and consistently measure the underlying construct of xerostomia symptoms. This robustness in internal consistency across different language adaptations strengthens the validity of the HXI as a reliable tool for assessing xerostomia.

As can be seen in Table 6, most studies did not report detailed factor analysis, which highlights the thoroughness of the validation process of the HXI. In line with the original factor analysis conducted by Thomson et al. [6], the HXI demonstrated a robust unidimensional structure. The HXI explained 80.3% of the variance, greater than the significantly lower variance explained in other full-length validations like the French version (32.7%) [23]. This high level of explained variance in the HXI underscores its strong unidimensional structure and its potential robustness in capturing the construct of xerostomia symptoms effectively.

### Strength and limitations

The main strength of the study includes the strict protocol utilized to collect and analyze the data. A limitation of this study is the use of a clinical convenience sample from a tertiary center, which may not be fully representative of the general population and could limit the generalizability of the findings to the entire population. Furthermore, it must be noted that the average HXI scores reported in this study are specific to our study cohort, and as such like other reported XI averages in the literature, these values may not be generalizable to the broader population or different clinical subgroups.

Our stimulated salivary flow results were obtained via a single stimulus rather than the continuous, interval-based stimulation described by Navazesh et al.^13^ This alternative approach was chosen to prioritize patient comfort and prevent irritation of the dry oral mucosa, consistent with our previously published methodology [33]. We acknowledge that this results in lower flow rates than with continuous stimulation, and comparisons should be made with studies using similar methods.

## Conclusions

The overall findings of reliability (Cronbach’s α) and validity (construct and discriminant validity), along with the comparative data for the HXI for other languages, confirm that the HXI is a valid and reliable instrument for assessing patient-reported oral dryness in Hebrew-speaking populations. Factor analyses, explaining 80.4% of the variance, confirm its unidimensional structure. The HXI can be used to support diagnosis, treatment, and evaluation of interventions in these populations. The HXI validation enables global multicenter trials, ensures methodological consistency, and facilitates scientific benchmarking across diverse linguistic backgrounds.

## Data Availability

All data supporting the findings of this study are available within the paper and its Supplementary Information.

## Appendix

**Table 7:** The Hebrew version of the Xerostomia Inventory (HXI) questionnaire

*שאלון להערכתXerostomia (יובש פה)*					
תמיד	לעיתים קרובות	לפעמים	לעיתים רחוקות	אף פעם	1. אני גומע נוזלים לסייע בבליעת מזון
					2. הפה שלי מרגיש יבש בעת אכילת ארוחה
					3. אני קם בלילה כדי לשתות
					4. הפה שלי מרגיש יבש
					5. יש לי קושי באכילת מזונות יבשים
					6. אני מוצץ סוכריות מתוקות או סוכריות שיעול להקל על יובש הפה
					7. יש לי קשיים לבלוע מזונות מסוימים
					8. עור הפנים שלי מרגיש יבש
					9. העיניים שלי מרגישות יבשות
					10. השפתיים שלי מרגישות יבשות
					11. פנים האף שלי מרגיש יבש

**Table 8:** Discriminant validity is presented by the associations of the total HXI score and sociodemographic variables (mean±SD using non-paired T test for two categories, ANOVA for three or more categories)

Variable	Category	Mean HXI score±standard deviation (SD)	*P* value
Sex	Men	40.1±13.4	0.959
	Women	39.9± 12.7	
Current work	Yes	39.1 ± 12.6	0.596
	No	40.5± 12.9	
Collar	White	36.4±13.0	0.286
	Blue	42.3± 11.9	
	Not Working	40.3± 12.9	
Birth country	Israel	39.5± 13.2	0.488
	Western Europe	42.3± 11.0	
	Eastern Europe	30.0 ±0.0	
	Former Soviet Union	38.0 ± 12.8	
	Asia	40.2± 13.3	
	Africa	41.2± 12.7	
	North America	44.5± 9.9	
	South America	13.0± 0.0	
Father birth country	Israel	40.8±13.6	0.981
	Western Europe	40.4±13.0	
	Eastern Europe	39.8±12.5	
	Former Soviet Union	37.4±12.9	
	Asia	38.4±13.0	
	Africa	41.3±12.8	
	North America	42.3±5.8	
	South America	33.5±28.9	
Mother birth country	Israel	40.7±11.8	0.456
	Western Europe	38.0±12.9	
	Eastern Europe	43.4±11.7	
	Former Soviet Union	36.7±12.7	
	Asia	38.8±13.5	
	Africa	39.9±13.9	
	North America	43.5±7.7	
	South America	13.0±0.0	

## Abbreviations

HXI: Hebrew XI validated version
PROM: Patient-reported outcome measures
QoL: Quality of life
sSjD: Secondary Sjögren’s disease
SjD: Sjögren’s disease
SE: Standard error
SSF: Stimulated salivary flow
XI: Xerostomia Inventory
XQ: Xerostomia Questionnaire
XeQoLS: Xerostomia Quality of Life Scale
